# Association between years with incidence of communicable diseases focused on COVID-19 and hand hygiene among adults in South Korea: a cross-sectional study

**DOI:** 10.1186/s12889-022-13951-x

**Published:** 2022-08-10

**Authors:** Yun Hwa Jung, Yu Shin Park, Eun-Cheol Park, Sung-In Jang

**Affiliations:** 1 Department of Public Health, Graduate School, Yonsei University, 03722 Seoul, Republic of Korea; 2 Institute of Health Services Research, Yonsei University, Seoul, 03722 Republic of Korea; 3 Department of Preventive Medicine, Yonsei University College of Medicine, 50 Yonsei-ro, Seodaemun-gu, 03722 Seoul, Republic of Korea

**Keywords:** COVID-19, Coronavirus disease, Communicable diseases, Hand disinfection, Hand hygiene

## Abstract

**Background:**

Handwashing is important considering the impact of communicable diseases on the public. We aimed to identify the association between years with incidence of communicable diseases during the coronavirus disease 2019 (COVID-19) pandemic and hand hygiene in South Korea.

**Methods:**

This cross-sectional study evaluated 5 years (2013, 2015, 2017, 2019, and 2020) of data from the Korea Community Health Survey and included 1,034,422 adults. Multinomial logistic regression analysis was performed to assess handwashing frequency by year. Receiver operating characteristic analysis was used to determine the cut-off point for handwashing frequency.

**Results:**

The always/frequently handwashing rate was 44.7%. This tendency was stronger in adults with each ascending year, with reference to 2013 (2015, odds ratio [OR] = 1.10, 95% confidence interval [CI] = 1.08, 1.13; 2017, OR = 1.10, 95% CI = 1.08, 1.13; 2019, OR = 1.17, 95% CI = 1.14, 1.20; 2020, OR = 3.21, 95% CI = 3.14, 3.29). Among women, the OR of frequently/always handwashing was 3.55 times higher (95% CI = 3.45, 3.66) in 2020 than in 2013. This OR was 2.95 among men (95% CI = 2.86, 3.04). In influenza-vaccinated participants, the OR of frequent/always handwashing was 3.25 times higher in 2020 than in 2013 (95% CI = 3.15, 3.36), while in non-vaccinated participants it was 3.17 (95% CI = 3.08, 3.27). Among adults who practiced physical distancing during the COVID-19 pandemic, the OR was 1.36 times higher (95% CI = 1.29, 1.42) with frequent handwashing, 1.64 times higher (95% CI = 1.57, 1.70) than those who did not practice it.

**Conclusions:**

There was a strong tendency toward frequent handwashing over the years; the trend was even greater in 2020 during the COVID-19 pandemic. Given that communicable diseases and handwashing are closely related, it is necessary to promote hand hygiene for prevention.

**Supplementary Information:**

The online version contains supplementary material available at 10.1186/s12889-022-13951-x.

## Background

The incidence of communicable diseases designated by law has tended to increase since 2013 in Korea. The total incidence per 100,000 people was 148.4 in 2013, 185.7 in 2015, 295.5 in 2017, 307.7 in 2019, and 281.6 in 2020. The most common communicable diseases were Middle East respiratory syndrome (MERS) in 2015, scarlet fever in 2017, viral hepatitis A in 2019, and coronavirus disease 2019 (COVID-19) in 2020 [1].

By August 2021, the cumulative confirmed number of COVID-19 cases in Korea was 251 421 accounting for 0.49% of the total population [2, 3]. The cumulative confirmation rates of COVID-19 cases were 11.56% in the United States, 9.86% in the United Kingdom, and 2.35% in India [4–7]. COVID-19 emerged as a novel coronavirus pneumonia in December 2019 and was declared a pandemic by the World Health Organization in March 2020. COVID-19 has an incubation period of 2–14 days, which is longer than that of flu (1–4 days) [8, 9]. In addition, unlike MERS, COVID-19 is highly contagious because the cell surface binding force is 10 times higher since the spike protein is activated by furin in the liver, lung, and small intestine [10].

Handwashing is effective in preventing communicable diseases. Proper handwashing can reduce the spread of transmissible diseases by 24%–31% and water- and food-borne communicable diseases by 50%–70% [11–13]. Handwashing under running water allows access to uncontaminated water [14]. The use of soap serves as a surfactant cleaner that removes dirt and microbes from the skin. In a previous study, handwashing after using public transport and public places with only water and with soap reduced bacteria from 44 to 23% and 8%, respectively [15]. Additionally, rubbing hands can remove dirt, grease, and microbes from the skin by creating friction, and washing for 15–30 s removes more germs [16].

The awareness rate of proper handwashing among Korean adults was high at 90.0%. However, the practice rate of proper handwashing was low at 72.4% [17]. Previous studies have shown that people tend to exaggerate how often they wash their hands [18]. Handwashing is the most basic way to prevent communicable diseases and is highly valuable in communicable disease prevention and control policy [19]. Understanding the importance of handwashing is crucial considering the impact of communicable diseases on the public.

Public handwashing may be related to historical and cultural factors in health and hygiene. Until the 1390 s in Korea, as Buddhism was transmitted as a precept, a culture that cares about hygiene was popularly developed. Thereafter, until 1910, a culture of partial washing of the body developed, and body exposure was considered taboo owing to Confucianism [20]. According to a survey by the Korea Development Institute in 1987, 69% of the respondents thought that the public’s concept of hygiene would increase. This seems to be related to the improvement of public sanitation facilities and the expansion of health and sanitation services for the 1988 Seoul Olympic Games. In 2005, the Korean government held a nationwide handwashing campaign with the participation of government agencies, medical circles, hygiene-related groups, civic groups, and educational groups [21]. However, this nationwide handwashing campaign was halted in 2014 because of budget issues [22]. With the outbreak of the COVID-19 pandemic, the disease-preventing effect of handwashing is being emphasized again.

The influence of the prevalence of communicable diseases in society to handwashing behavior may be related to the health belief model (HBM) or social learning theory. HBM is a representative theory that explains the intention of an individual’s health behavior and can be applied to understanding infection prevention behavior such as COVID-19 vaccination and handwashing. The components of HBM in the context of handwashing according to the prevalence of communicable diseases are as follows [23]. As a perceived susceptibility factor, it can be thought that the prevalence of communicable diseases can lead to infection. An example of a perceived severity factor is that an infected person will have a life or health restriction. As a perceived benefit factor, it may be thought that handwashing helps prevent infection with communicable diseases. As perceived barriers to action factors, handwashing can lead to skin itching and dryness, which can be inconvenient. As a cue to action factor, information about handwashing for the prevention of communicable diseases have been shown in the news. Furthermore, as a self-efficacy factor, people who think they can perform handwashing to prevent infectious diseases can take action. In social learning theory, human, behavioral, and environmental factors interact with each other [24]. People can learn proper handwashing and hygiene practices by observing and mimicking good handwashing behavior in society.

This study aims to contribute to the prevention of public infection by understanding social handwashing in relation to communicable diseases. By focusing on the period when there are no significant changes in the campaigns or accessibility, it would be possible to understand directly the association between the incidence of communicable diseases and handwashing. In particular, we focused on handwashing in 2020 when COVID-19 was prevalent. Four common items of the handwashing questionnaire were analyzed over the 5-year investigation, and we investigated handwashing in terms of COVID-19-related variables.

## Methods

### Data

The research data were obtained from the Korea Community Health Survey (KCHS) of 2013, 2015, 2017, 2019, and 2020. KCHS has been performing surveys on handwashing, which is the dependent variable of this study, every two years since 2013. KCHS performed a survey on handwashing in 2020 as an exception because of the COVID-19 pandemic. The KCHS is a representative anonymous, self-reported online survey of Korean adults conducted by the Korea Disease Control and Prevention Agency (KDCA). Since 2008, the KDCA has been conducting a nationwide survey every year. KDCA published the KCHS Data Profiles as a brief report in 2015 to describe the data [25]. The survey for our study was conducted from August 2020 to October 2020. In this study, stratified multi-stage cluster sampling was performed using the National Census Registry of variables for household and 17 individual survey coverages [26]. On the basis of geographic and demographic distribution, participants’ data were weighted and generalizable to Korean nationals [27]. There were 142 questions regarding socioeconomic status, health behavior, health education, and health screening [28]. The present study was approved by the institutional review board (IRB) of Yonsei University's Health System (IRB number: Y-2020-0031). 

### Participants

Participants were adults aged 19 years or older. A total of 1,034,422 adults participated in the study, consisting of 480,923 men and 553,499 women. Among the participants, 109,666 persons with uncertain responses were excluded because of missing data.

### Variables

The variables of interest were the years 2013, 2015, 2017, 2019, and 2020. The dependent variable, handwashing, was investigated in only the aforementioned years. The dependent variable was the total score of handwashing calculated by a questionnaire including four common questions during the survey years. The questionnaire consisted of contents related to the investigation of previous studies [29–32]. The handwashing behavior questions were about handwashing “before eating”, “after toilet use”, “after going out”, and “with sanitizer”, and scores were assigned from four points. Four points indicated always washing hands and one point indicated never washing hands. As a result of the receiver operating characteristic analysis based on the handwashing states, in which all responses to the questions were handwashing frequently or always, the area under curve was 0.931, sensitivity was 0.699, and specificity was 1.000 for a cut-off point of 14.999. Therefore, we selected 15 points as the cut-off score to distinguish whether participants washed their hands frequently. The scale was categorized as 0–14 points (not handwashing always/frequently), 15 points (handwashing frequently), and 16 points (handwashing always).

The covariates were demographic variables (sex and age), socioeconomic variables (marital status, region, household income, occupational categories, and educational level), a variable related to mental health (perceived stress), variables related to health behavior (current drinking and current smoking status), and medical utilization (unmet medical need, influenza vaccination, self-perceived health status).

### Statistical analysis

To evaluate the association between years with the incidence of communicable diseases during the COVID-19 pandemic and hand hygiene, we conducted multiple logistic regression analysis using the PROC SURVEYLOGISTIC procedure with weight, cluster, and strata for analysis. Results included odds ratios (ORs) and 95% confidence intervals (95% CIs). For all variables, there was no multicollinearity using the variance inflation factor. To investigate the dose–response relationship, multinomial logistic regression was also performed. Results from analysis of variance and tests of independence were also valid. The trend test was conducted to identify the relationship between the independent and dependent variables. Statistical analysis was performed using SAS, version 9.4 (SAS Institute Inc., Cary, NC).

## Results

Table 1 indicates the general characteristics of the participants. Among the 1,034,422 adults, the sex ratio was similar, with 480,923 men (46.5%) and 553,499 women (53.5%). The mean age of adults was 55.0 ± 16.9 years. The prevalence of handwashing always/frequently increased over the years (2013: 36.6%, 2015: 39.5%, 2017: 40.1%, 2019: 40.1%, 2020: 64.9%).

**Table 1:** General characteristics of the study population ^a^

Variables	Handwashing						
	**Total**		**Yes**		**No**		***P-value***
	**N**	**%**	**N**	**%**	**N**	**%**	
**Total (*N* = 1,034,422)**	1,034,422	100.0	462,123	44.7	572,299	55.3	
**Year**							< 0.0001
2013	187,837	18.2	68,670	36.6	119,167	63.4	
2015	225,086	21.8	88,855	39.5	136,231	60.5	
2017	225,174	21.8	90,324	40.1	134,850	59.9	
2019	173,705	16.8	69,712	40.1	103,993	59.9	
2020	222,620	21.5	144,562	64.9	78,058	35.1	
**Sex**							< 0.0001
Men	480,923	46.5	182,960	38.0	297,963	62.0	
Women	553,499	53.5	279,163	50.4	274,336	49.6	
**Age (mean: 55.0, SD: 16.9)**							< 0.0001
19–29	92,868	9.0	48,713	52.5	44,155	47.5	
30–39	133,009	12.9	75,886	57.1	57,123	42.9	
40–49	183,532	17.7	92,431	50.4	91,101	49.6	
50–59	211,160	20.4	96,030	45.5	115,130	54.5	
60–69	189,775	18.3	80,490	42.4	109,285	57.6	
70-	224,078	21.7	68,573	30.6	155,505	69.4	
**Marital status**							< 0.0001
Living with spouse	712,644	68.9	325,059	45.6	387,585	54.4	
Living without spouse	321,778	31.1	137,064	42.6	184,714	57.4	
**Region**							< 0.0001
Urban area	298,002	28.8	155,510	52.2	142,492	47.8	
Rural area	736,420	71.2	306,613	41.6	492,807	58.4	
**Household income**							< 0.0001
High	300,755	29.1	156,908	52.2	143,847	47.8	
Mid	336,671	32.5	159,020	47.2	177,651	52.8	
Low	396,996	38.4	146,195	36.8	250,801	63.2	
**Occupational categories **^**b**^							< 0.0001
White collar	202,879	19.6	114,900	56.6	87,979	43.4	
Pink collar	138,382	13.4	71,784	51.9	66,598	48.1	
Blue collar	336,648	32.5	120,545	35.8	216,103	64.2	
Inoccupation	356,513	34.5	154,894	43.4	201,619	56.6	
**Educational level**							< 0.0001
Middle school or less	375,346	36.3	122,086	32.5	253,260	67.5	
High school	300,958	29.1	139,165	46.2	161,793	53.8	
College or over	358,118	34.6	200,872	56.1	157,246	43.9	
**Perceived stress**							< 0.0001
Much	246,677	23.8	112,589	45.6	134,088	54.4	
Less	787,745	76.2	349,534	44.4	438,211	55.6	
**Current drinking**							< 0.0001
Yes	670,820	64.8	304,360	45.4	366,460	54.6	
No	363,602	35.2	157,763	43.4	205,839	56.6	
**Current smoking**							< 0.0001
Yes	187,325	18.1	69,082	36.9	118,243	63.1	
No	847,046	81.9	393,011	46.4	454,035	53.6	
**Unmet medical need**							< 0.0001
No	936,265	90.5	425,249	45.4	511,016	54.6	
Yes	98,157	9.5	36,874	37.6	61,283	62.4	
**Influenza vaccination**							< 0.0001
Yes	541,140	52.3	237,704	43.9	303,436	56.1	
No	493,282	47.7	224,419	45.5	268,863	54.5	
**Self-perceived health status**							< 0.0001
Bad	204,780	19.8	63,679	31.1	141,101	68.9	
Normal	429,471	41.5	189,882	44.2	239,589	55.8	
Good	400,171	38.7	208,562	52.1	191,609	47.9	

^a^ Table 1 shows the results of univariate analyses that examined between years with the incidence of communicable diseases focused on COVID-19 pandemic and washing hands. p ≤ 0.05 was considered statistically significant

^b^ Three groups (white, pink, and blue) based on the international standard classification occupations codes

Table 2 presents the factors associated with handwashing. Adults who were handwashing always had ORs that gradually increased by year with reference to 2013 (2015, OR = 1.10, 95% CI = 1.08–1.13; 2017, OR = 1.10, 95% CI = 1.08–1.13; 2019, OR = 1.17, 95% CI = 1.14–1.20; 2020, OR = 3.21, 95% CI = 3.14–3.29). In the results of the linear hypotheses testing, a “*P* for trend < 0.0001” was observed. Specifically, the OR for handwashing frequently was 2.49 times higher (95% CI = 2.42–2.56) in 2020 than in 2013. Concerning handwashing always, this OR was 3.61 times higher (95% CI = 3.51–3.71) in 2020 than in 2013 (Supplementary Fig. 1).

**Table 2:** Results of factors associated with handwashing ^a^

Variables	Handwashing			
	**OR**	**95% CI**		
**Year **^**a**^				
2013	1.00			
2015	1.10	(1.08	–	1.13)
2017	1.10	(1.08	–	1.13)
2019	1.17	(1.14	–	1.20)
2020	3.21	(3.14	–	3.29)
**Sex**				
Men	1.00			
Women	1.93	(1.90	–	1.96)
**Age**				
19–29	1.00			
30–39	1.24	(1.21	–	1.27)
40–49	1.07	(1.04	–	1.09)
50–59	1.02	(0.99	–	1.04)
60–69	1.09	(1.06	–	1.12)
70-	0.78	(0.76	–	0.80)
**Marital status**				
Living with spouse	1.20	(1.18	–	1.21)
Living without spouse	1.00			
**Region**				
Urban area	1.23	(1.21	–	1.24)
Rural area	1.00			
**Household income**				
High	1.05	(1.03	–	1.07)
Mid	1.04	(1.02	–	1.05)
Low	1.00			
**Occupational categories**^**a**^				
White collar	1.17	(1.15	–	1.19)
Pink collar	1.09	(1.07	–	1.11)
Blue collar	0.91	(0.89	–	0.92)
Inoccupation	1.00			
**Educational level**				
Middle shool or less	1.00			
High school	1.50	(1.47	–	1.53)
College or over	1.99	(1.95	–	2.03)
**Perceived stress**				
Much	1.00			
Less	0.94	(0.93	–	0.96)
**Current drinking**				
Yes	1.00			
No	1.12	(1.10	–	1.13)
**Current smoking**				
Yes	1.00			
No	1.12	(1.10	–	1.14)
**Unmet medical need**				
No	1.21	(1.18	–	1.23)
Yes	1.00			
**Influenza vaccination**				
Yes	1.31	(1.29	–	1.33)
No	1.00			
**Self-perceived health status**				
Bad	1.00			
Normal	1.21	(1.19	–	1.23)
Good	1.49	(1.46	–	1.52)

^a^ Table 2 shows that adults who were washing hands always had gradually increased ORs by years referring to 2013. *P* for trend < 0.0001; odds ratio (OR); 95% confidence intervals (95% CI)

Table 3 shows the results of subgroup analysis stratified by years. Subgroup analysis of handwashing always and frequently was performed with reference to 2013. Women and those vaccinated against influenza were more likely to wash hands frequently/always, especially in 2020 compared to 2013 than each counterpart (men and those not vaccinated against influenza) (women, OR = 3.55, 95% CI = 3.45–3.66; men, OR = 2.95, 95% CI = 2.86–3.04; those vaccinated against influenza, OR = 3.25, 95% CI = 3.15–3.36; those not vaccinated against influenza, OR = 3.17, 95% CI = 3.08–3.27).

**Table 3:** Results of subgroup analysis stratified by independent variables ^a, b^

Variables	Handwashing																
	**Year**																
	**2013**	**2015**				**2017**				**2019**				**2020**			
	**OR**	**OR**	**95% CI**			**OR**	**95% CI**			**OR**	**95% CI**			**OR**	**95% CI**		
**Sex**																	
Men	1.00	1.07	(1.04	–	1.10)	1.08	(1.04	–	1.11)	1.14	(1.11	–	1.18)	2.95	(2.86	–	3.04)
Women	1.00	1.13	(1.10	–	1.16)	1.12	(1.09	–	1.15)	1.21	(1.17	–	1.25)	3.55	(3.45	–	3.66)
**Influenza vaccination**																	
Yes	1.00	1.14	(1.10	–	1.17)	1.12	(1.09	–	1.16)	1.20	(1.16	–	1.24)	3.25	(3.15	–	3.36)
No	1.00	1.08	(1.05	–	1.11)	1.09	(1.06	–	1.12)	1.15	(1.11	–	1.18)	3.17	(3.08	–	3.27)

^a^ Reference group: No financial decline (perceived household financial decline due to COVID-19); odds ratio (OR); 95% confidence intervals (95% CI)

^b^ Adjusted for other covariates

In Table 3, subgroup analysis was performed by independent variables. Women and individuals vaccinated against influenza were more likely to wash their hands frequently/always, especially in 2020.

Table 4 presents the results of interaction factors analysis associated with handwashing. Good self-perceived health status in 2020 had a higher OR in terms of handwashing than bad self-perceived health status in 2017. The OR of handwashing after going out was 1.11 times higher (95% CI = 1.08–1.15) among those who self-perceived their health status as good in 2020 than among those who self-perceived it as bad in 2013. The OR of handwashing with soap was 1.05 times higher (95% CI = 1.03–1.07) for the same. The OR of handwashing after going out was the highest in 2020 compared with that in other years, especially the OR of handwashing always (2015, OR = 1.14, 95% CI = 1.07–1.23; 2017, OR = 1.27, 95% CI = 1.18–1.36; 2019, OR = 1.89, 95% CI = 1.75–2.03; 2020, OR = 17.32, 95% CI = 15.35–19.55). Moreover, the OR of handwashing always with soap was the highest in 2020 compared to that in 2013, 2015, 2017 and 2019 (2015, OR = 1.13, 95% CI = 1.08–1.19; 2017, OR = 1.15, 95% CI = 1.09–1.21; 2019, OR = 1.03, 95% CI = 0.97–1.08; 2020, OR = 6.51, 95% CI = 6.06–7.01) (Supplementary Fig. 2).

**Table 4:** Results of interaction factors associated with handwashing ^a, b^

**Variables**	**Handwashing**																			
	**Washing frequently**				**Washing before eating**				**Washing after toilet**				**Washing after going out**				**Washing with soap**			
	**OR**	**95% CI**			**OR**	**95% CI**			**OR**	**95% CI**			**OR**	**95% CI**			**OR**	**95% CI**		
**Year**																				
2013	1.00				1.00				1.00				1.00				1.00			
2015	0.82	(0.81	–	0.83)	0.83	(0.81	–	0.84)	0.76	(0.74	–	0.78)	0.63	(0.62	–	0.64)	0.81	(0.80	–	0.82)
2017	0.83	(0.82	–	0.84)	0.81	(0.80	–	0.83)	0.80	(0.78	–	0.82)	0.67	(0.66	–	0.68)	0.80	(0.79	–	0.82)
2019	0.89	(0.87	–	0.90)	0.76	(0.75	–	0.78)	0.79	(0.77	–	0.81)	0.74	(0.73	–	0.76)	0.80	(0.78	–	0.81)
2020	2.18	(2.15	–	2.21)	2.34	(2.28	–	2.40)	2.75	(2.66	–	2.84)	4.95	(4.79	–	5.12)	2.69	(2.63	–	2.74)
**Self-perceived health status**																				
Bad	1.00				1.00				1.00				1.00				1.00			
Good	1.13	(1.12	–	1.13)	1.20	(1.19	–	1.22)	1.16	(1.15	–	1.17)	1.16	(1.15	–	1.17)	1.10	(1.09	–	1.11)
**Year x Self-perceived health status**																				
2013 × Bad	1.00				1.00				1.00				1.00				1.00			
2015 × Good	0.98	(0.97	–	0.99)	0.97	(0.96	–	0.99)	0.95	(0.93	–	0.97)	0.95	(0.94	–	0.97)	0.98	(0.97	–	1.00)
2017 × Good	0.99	(0.98	–	1.00)	0.98	(0.96	–	0.99)	0.98	(0.96	–	1.00)	0.97	(0.95	–	0.98)	0.98	(0.97	–	0.99)
2019 × Good	1.00	(0.99	–	1.01)	0.99	(0.97	–	1.01)	0.97	(0.95	–	0.99)	0.98	(0.96	–	1.00)	0.98	(0.97	–	1.00)
2020 × Good	1.01	(1.00	–	1.02)	1.08	(1.06	–	1.10)	1.09	(1.06	–	1.13)	1.11	(1.08	–	1.15)	1.05	(1.03	–	1.07)

^a^ Reference group: not washing hands always/frequently, not washing hands before eating / after toilet / after outing / with soap; odds ratio (OR); 95% confidence intervals (95% CI)

^b^ Adjusted for other covariates

Table 4 shows the changes in handwashing behaviors by situation according to years and self-perceived health status.

**Fig. 1:**
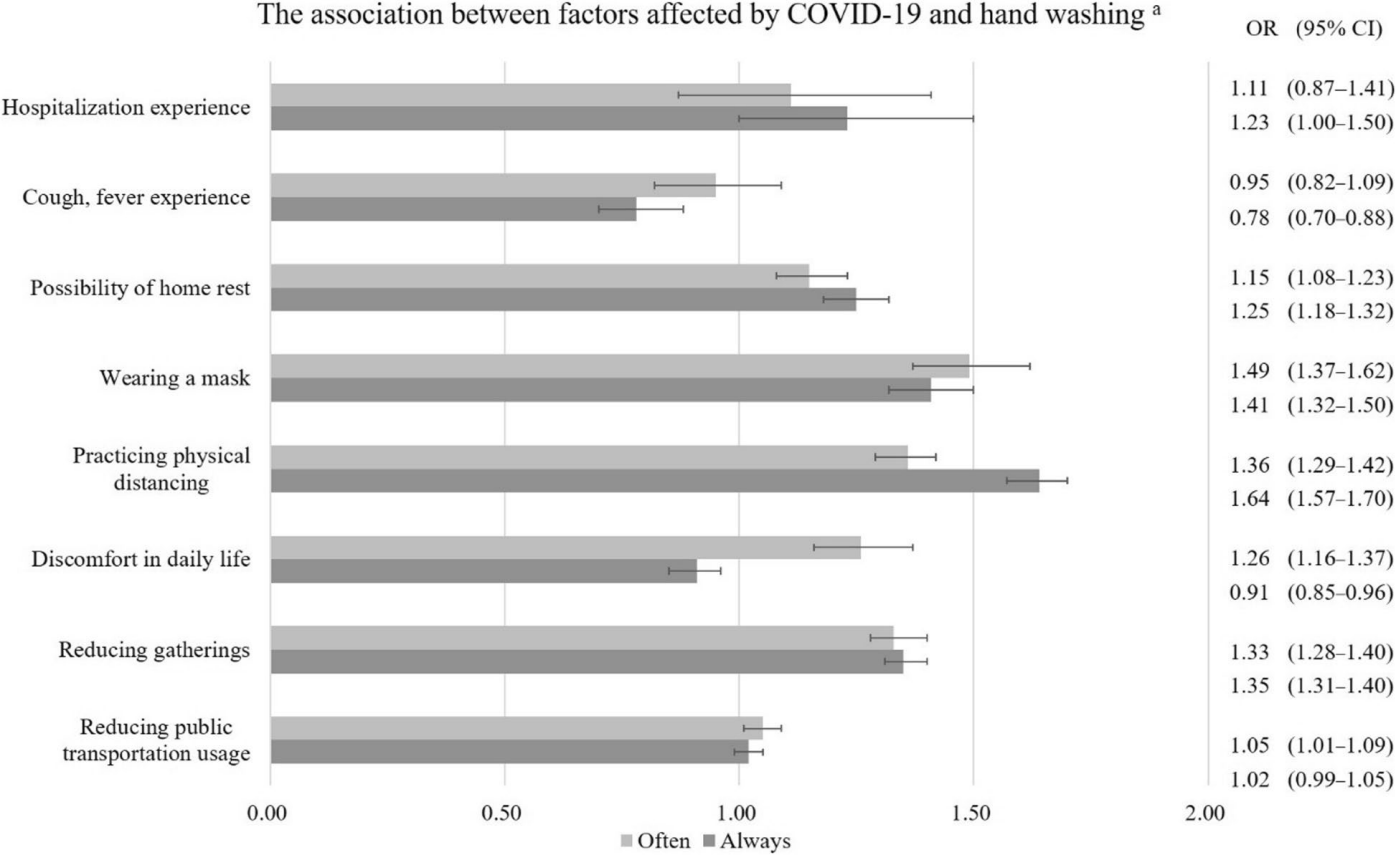
The association between factors affected by COVID-19 and handwashing ^a, b^. ^a^ Reference group: unaffected by each factor; odds ratio (OR); 95% confidence intervals (95% CI). ^b^ Adjusted for other covariates

Figure 1 indicated the association between health behavior factors affected by COVID-19 and handwashing. Regarding the results of the handwashing questions, the most influential practice affected by COVID-19 was practicing physical distancing. Adults who practiced physical distancing because of COVID-19 had 1.36 times higher OR with frequent handwashing (95% CI = 1.29–1.42) than otherwise. These adults had 1.64 times higher OR of always handwashing (95% CI = 1.57–1.70) than those who were not practicing physical distancing.

Regarding the results of the handwashing questions, Fig. 1 shows the impact of COVID-19 on handwashing.

## Discussion

As the years passed, the frequency of handwashing always/frequently increased among adults, especially in 2020, the COVID-19 pandemic era. The tendency was evident in the following cases: women and those vaccinated against influenza. The frequencies of handwashing after going out and handwashing with soap tended to increase in 2020 when self-perceived health status was good compared with when it was poor in 2013. Those practicing physical distancing due to COVID-19 were more likely to wash their hands always/frequently.

In this 5-year survey, 44.7% of adults responded that they wash their hands always/frequently, while the rest said they did not. This is consistent with findings of previous studies that fewer people wash their hands always/frequently in the absence of special events, such as pandemics or handwashing campaigns [17, 30, 33].

There is a very close positive linear relationship between years with the incidence of communicable diseases and handwashing. This is especially evident in 2020 during the COVID-19 pandemic. As the incidence of communicable diseases mostly affected public awareness in a specific year, the likelihood of handwashing always/frequently was high. In previous empirical surveys, the factors influencing handwashing behavior included campaigns, handwashing accessibility, and health beliefs [34]. In Korea, campaigns and handwashing accessibility did not change significantly during the survey period used in this study (2013, 2015, 2017, 2019, and 2020). Since 2014, the Korean government has not implemented a nationwide long-term handwashing campaign. The number of public toilets nationwide was 58,248 in 2014 [35] and 56,451 in 2020 [36], which is equivalent to 1 toilet per 1,000 people.

Additionally, handwashing can be affected by the severity of the communicable diseases in terms of their causes, period of the epidemic, age distribution of infection, the incidence rate in residential areas, and mortality. The incidence of communicable diseases designated by law per 100,000 people was low (148.4) in 2013, and tended to increase thereafter. The incidence in 2015 was 185.7 people. MERS accounted for 0.14% of the total communicable diseases, but it had an impact on public awareness because a high fatality rate (20.5%) was recorded within a short period (46 days) in areas with high incidence (Seoul & Gyeonggi, regional incidence: 63.2%) [2, 37]. The incidence of communicable diseases designated by law per 100,000 people in 2017 was 295.5, and scarlet fever accounted for 12.5% of the total communicable diseases. The incidence of scarlet fever increased by 91.7% compared with that in the previous year, and it affected certain age groups (3–6 years, incidence by age: 71.5%) and regions (Gyeonggi, regional incidence: 30.1%, ranking of the regional incidence by population: first) [2, 38]. The incidence of communicable diseases designated by law per 100,000 people in 2019 was 307.7 people, and viral hepatitis A accounted for 9.5% of the total communicable diseases. The incidence of viral hepatitis A increased by 622.1% compared with that in the previous year because of a specific cause (the consumption of contaminated shellfish) in certain age groups (30–40 years, incidence by age: 72.7%) and region (Gyeonggi, regional incidence: 30.7%; Daejeon, ranking of the regional incidence by population: first) [2, 39]. The incidence of communicable diseases designated by law per 100,000 people in 2020 was 281.6 people, and COVID-19 cases accounted for 35.4% of the total. COVID-19 affected a specific period (December, monthly incidence: 45.0%), age group (from fifties to sixties, incidence by age: 35.4%), and regions (Seoul & Gyeonggi regional incidence: 57.4%, ranking of the regional incidence by population: first) [1, 2, 40].

The incidence of communicable diseases increased by year, and women were more likely to wash their hands always/frequently compared with men. This is consistent with results of previous studies showing that women have better hand hygiene than men [30, 41, 42]. Sex can affect severity and individual susceptibility to disease [43]. This may help women become more aware of and prevent communicable diseases. In previous studies, women were more likely to perceive H1N1 influenza infection as fatal [43]. Furthermore, women were more likely to follow recommendations for preventing H1N1 influenza, severe acute respiratory syndrome, and other communicable diseases than men [41, 44]. People vaccinated against influenza were more likely to hand wash frequently/always in years with a high incidence of communicable diseases. This may be due to concerns about communicable diseases. Prior studies have shown that people worrying about risk take measures to reduce risk, and people who are concerned about communicable diseases try to follow preventive measures, such as handwashing [45]. The more people worried about seasonal influenza in France, the more likely they were to get the A/H1N1 influenza vaccine. Old age and the presence of chronic disease in Europe were closely related to vaccination [46, 47].

People with good self-perceived health status in 2020 were more likely to wash their hands always/frequently compared with those with bad self-perceived health status in 2013. The trend was particularly evident in handwashing after going out. People following physical distancing due to COVID-19 were also more likely to always wash their hands than those not following physical distancing. COVID-19 is a highly contagious disease, and our investigation was conducted before its vaccine was released; thus, there was high anxiety about infection among the public. COVID-19 is transmitted through respiratory droplet spread; therefore, individuals need to be careful when using public transportation or crowded facilities. Handwashing has been recommended by the government as a representative prevention method for communicable diseases.

To the best of the authors’ knowledge, this is the first study to identify an association between communicable diseases, focusing on COVID-19, and routine handwashing by years using national survey data of adults. Data from random cluster sampling are sufficiently representative of Korean adults.

The limitations of this study are as follows. First, the causality between communicable diseases, specifically COVID-19, and routine handwashing is obscure. As this study had a cross-sectional design, it was difficult to determine causality [48]. Nevertheless, while comparing the survey responses over 5 years, we assessed the change in handwashing behavior over time. Second, the specific frequency or duration of handwashing was not investigated. However, handwashing frequency and cases allow inference of its practice. Third, changes in handwashing related to communicable diseases in infants and adolescents are unknown. In previous studies, there were a few cases (after using the toilet/going out, before eating) of less handwashing among teenagers; therefore, further studies are needed [49, 50].

## Conclusions

Communicable diseases including COVID-19 are closely related to handwashing practices in adults, especially among women and those vaccinated against influenza. Adults are more likely to wash their hands when practicing physical distancing. Adults washed their hands with soap more frequently after going out after the outbreak of COVID-19 in 2020 than before. Given that communicable diseases and handwashing are closely related, it is necessary to promote hand hygiene for prevention. Proper handwashing is required for people at risk of contracting communicable diseases, especially COVID-19.

## Supplementary Information

**Additional file 1.**

## Data Availability

Publicly available datasets were analyzed in this study. These data can be found here: [https://chs.kdca.go.kr/chs/rdr/rdrInfoProcessMain.do] (accessed on 22 July 2022).

## Abbreviations

COVID-19: Coronavirus disease 2019
OR: Odds ratio
CI: Confidence interval
MERS: Middle East respiratory syndrome
KCHS: Korea Community Health Survey
KDCA: Korea Disease Control and Prevention Agency
