# Supplementary material for: Association between years with incidence of communicable diseases focused on COVID-19 and hand hygiene among adults in South Korea: a cross-sectional study
Source: BMC Public Health. 2022 Aug 10;22:1526. doi: 10.1186/s12889-022-13951-x (PMC9364310; doi:10.1186/s12889-022-13951-x)
Supplement: Supplementary file 1 — Additional file 1. [file 12889_2022_13951_MOESM1_ESM.docx]

**Supplementary figure 1.**


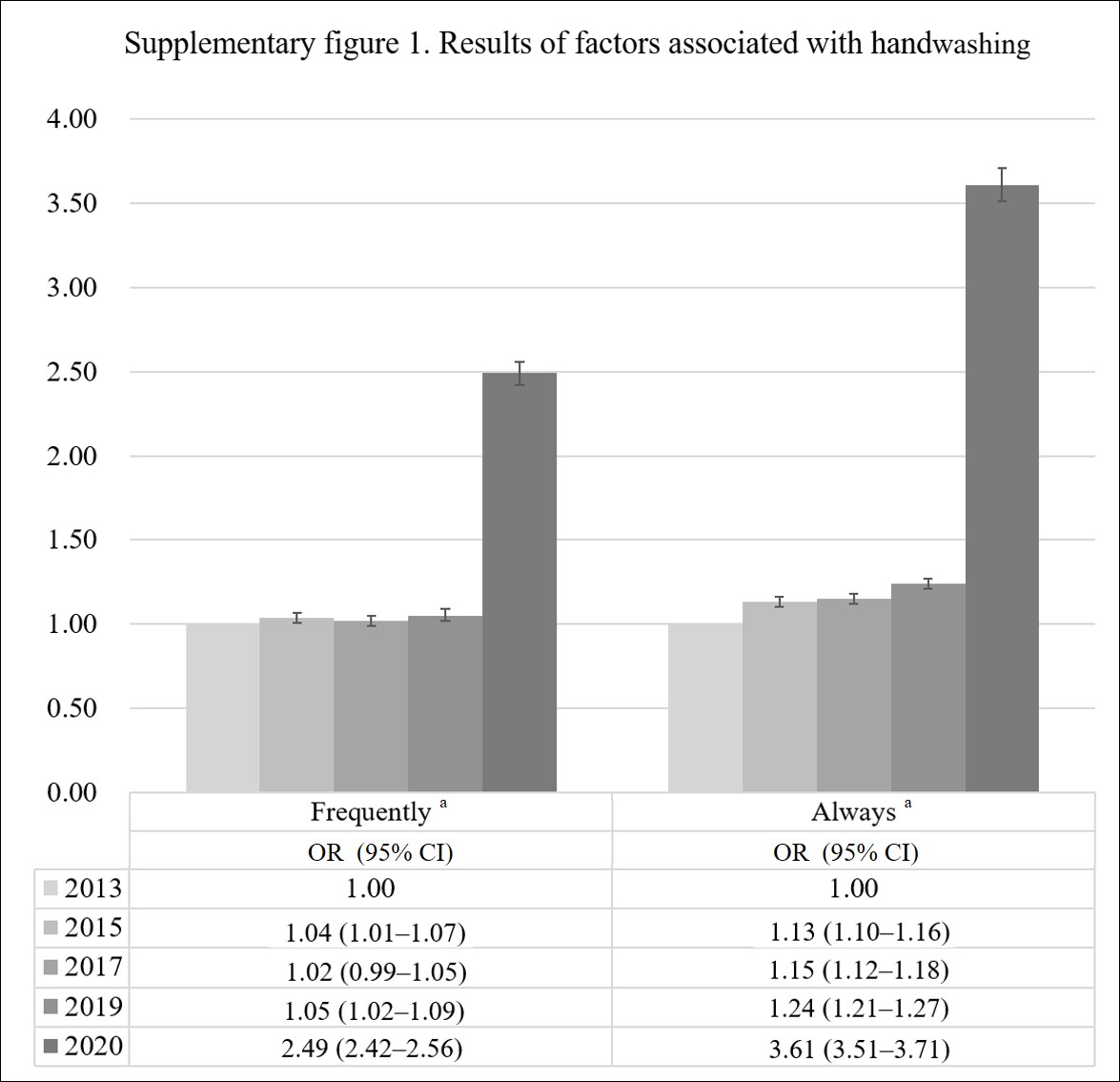


^a^ *P* for trend <0.0001; odds ratio (OR); 95% confidence intervals (95% CI)

Supplementary figure 1 showed the change in always handwashing by year 2013 as a reference.

Adjusted for other covariates

**Supplementary figure 2.**


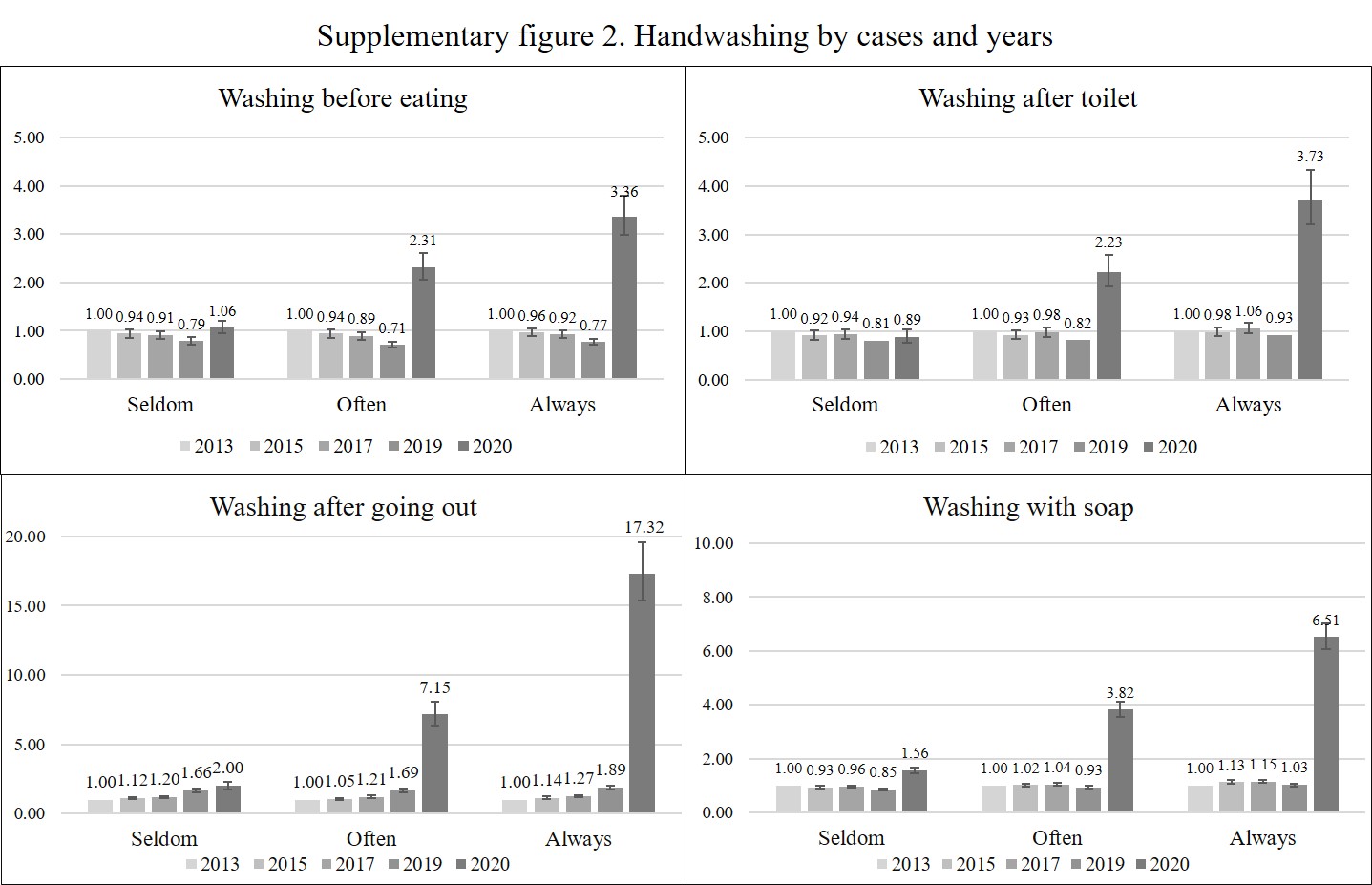


Supplementary figure 2 showed handwashing situations as odds ratios (numbers on the bars) and 95% confidence intervals (lines across the bars) by year and frequency.

Adjusted for other covariates
